# Manipulation of Innate and Adaptive Immunity through Cancer Vaccines

**DOI:** 10.1155/2017/3145742

**Published:** 2017-02-06

**Authors:** Elias J. Sayour, Duane A. Mitchell

**Affiliations:** UF Brain Tumor Immunotherapy Program, Preston A. Wells Jr. Center for Brain Tumor Therapy, Department of Neurosurgery, University of Florida, Gainesville, FL, USA

## Abstract

Although cancer immunotherapy has shown significant promise in mediating efficacious responses, it remains encumbered by tumor heterogeneity, loss of tumor-specific antigen targets, and the regulatory milieu both regionally and systemically. Cross talk between the innate and adaptive immune response may be requisite to polarize sustained antigen specific immunity. Cancer vaccines can serve as an essential fulcrum in initiating innate immunity while molding and sustaining adaptive immunity. Although peptide vaccines have shown tepid responses in a therapeutic setting with poor correlates for immune activity, RNA vaccines activate innate immune responses and have shown promising effects in preclinical and clinical studies based on enhanced DC migration. While the mechanistic insights behind the interplay between innate and adaptive immunity may be unique to the immunotherapeutic being investigated, understanding this dynamic is important to coordinate the different arms of the immune response in a focused response against cancer antigens.

## 1. Introduction

Immune targeting of cancer antigens has been employed since the original experiments of William H. Coley in the late 1800s [1]. In 1891, Coley administered streptococcal organisms into an inoperable patient and was able to demonstrate tumor regression [1]. He later injected over a thousand cancer patients with bacterial products (i.e.,* Streptococcus pyogenes*,* Serratia marcescens* known as Coley's toxins) and demonstrated promising results in both bone and soft tissue sarcomas [1, 2]. Coley's experiments utilized toll-like receptor (TLR) agonists in the bacterial products to harness the innate immune response [2]. Now, over a century later, cancer immunotherapy is a burgeoning field and has elicited statistically significant survival benefits in patients with refractory malignancies [3–5]. With the advent of sipuleucel-T and ipilimumab, the first FDA approved immunotherapeutics have arrived and the immune correlates for successful intervention are slowly being unraveled [3–5]. However, while cancer immunotherapy has shown significant promise in mediating efficacious response, it remains encumbered by tumor heterogeneity, loss of tumor-specific antigen targets, and the regulatory milieu both regionally and systemically [6–9]. Cancer vaccines have especially lagged behind checkpoint blockers and adoptive cellular therapy (ACT) in mediating robust antitumor immunity [10, 11].

Cancer vaccines deliver tumor antigens in the form of novel mutated epitopes, viral epitopes, developmental antigens, or self-differentiation antigens to endogenous innate cells (i.e., antigen presenting cells) [12–16]. Antigen presenting cells (APCs) become activated in the presence of vaccine carrying antigen, typically accompanied by adjuvants that provide the necessary inflammatory cues [17]. APCs then process the vaccine's tumor antigens into epitopes, which they present on the surface of their MHC class I and class II molecules for presentation to CD4 and CD8+ T cells [17]. They subsequently traffic to local draining lymph nodes, engaging, activating, and redirecting T cells to reject the presented tumor epitopes [17]. For a cancer vaccine to be effective, APC activation, trafficking, and T cell priming are essential initial steps. Secondary steps such as T cell proliferation, trafficking, tumor extravasation, and engagement of tumor antigens are also essential. In this review, we will examine promising strategies employed to enhance the initial steps of effective cancer vaccination followed by discussion of their synergistic roles with immune checkpoints to potentiate requisite secondary steps.

## 2. Initiating Innate Immunity

The innate immune system, as one of the first lines of defense, must recognize danger signals and respond accordingly [18]. Although T cells are the hallmark of an effective cancer immunotherapeutic response, they are often ineffective without activation of the innate immune system [10]. Innate immune cells such as natural killer cells, neutrophils, and macrophages have been shown to mediate regression in resistant murine models and can be harnessed in conjunction with adoptive T cell strategies [10, 19–21]. As opposed to discriminating between self and nonself, the danger theory purports that the innate immune system is primarily activated in response to danger signals [22, 23]. Pattern recognition receptors (PRRs) (i.e., endosomal TLRs and cytosolic sensors such as retinoic acid inducible gene I (RIGI) and melanoma differentiation-associated protein 5 (MDA5)) within innate immune cells are vital for perceiving danger signals (i.e., pathogen associated molecular patterns (PAMPs) and damage associated molecular patterns (DAMPs)) released from affected cells [18, 24, 25]. Since the kinetics of the immune response are sensitive to danger signals, methods to exploit this sensitivity may potentiate adaptive immunity [26]. This has been routinely employed through the use of chemotherapy such as cyclophosphamide and fludarabine in conjunction with promising T cell therapies including adoptively transferred tumor infiltrating lymphocytes (TILs) and chimeric antigen receptor (CAR) modified T cells [10, 27–30]. Although lymphodepletion with cyclophosphamide and fludarabine might be expected to mitigate against potent T cell responses, these agents have been associated with more potent cellular immunotherapy [31]. These enhancing effects are attributed not only to removal of regulatory compartments such as regulatory T cells (i.e., FoxP3+CD25+ T cells) and myeloid derived suppressor cell populations but also to released DAMPs which assist in engendering the inflammatory niche necessary to sustain the survival of adoptively transferred antigen specific T cells [31–35]. Although host-conditioning may be necessary to potentiate adoptive cell therapy, its implementation conflicts with immunotherapy's promise of delivering targeted agents without deleterious effects. Alternatively, development of experimental therapies that leverage both innate and adaptive immune arms without illicit effect is more attractive [36]. While synergy between innate and adaptive immunity is expected to potentiate immunotherapeutic response, chronic inflammatory stimuli may stymie an effective adaptive response [37–40]. In preclinical studies, low-dose infection with intradermal* Trypanosoma congolense* mediated expansion of regulatory T cells [37–40]. Similarly, as malignancies grow, they may precipitate similar levels of inflammation that predispose induction of regulatory cell subsets, which ultimately confound an appropriate adaptive response [34, 41, 42]. These inadequate innate responses predispose formation of regulatory cells that usurp effector T cells [34, 41, 42].

However, redirecting the innate immune system has become tenable locally (at the tumor site) with oncolytic viruses [43, 44]. Oncolytic viruses can be native or attenuated and be preferentially harnessed against cancer cells [43, 44]. Since cancer cells contain low levels of protein kinase R (regulates abnormal cell proliferation and antiviral response), oncolytic viruses can preferentially replicate within malignant tumors [43–45]. Preferential transfection and replication within tumor cells mediate their direct lysis, releasing both soluble tumor antigens and DAMPs which lead toward a broader systemic response against the tumor [43]. Similar to oncolytic viruses, cancer vaccines can be harnessed to incite an innate response against select tumor antigens but they bypass the complexity of intratumoral oncolytic viral administration by activating innate immunity peripherally.

## 3. Cancer Vaccines

Cancer vaccines can be harnessed to educate the senescent immune system against tumor antigens. DAMPs are released after local vaccine injection, allowing APCs to traffic, pick up tumor antigens, and migrate to local draining lymph nodes where they present their antigens to T cells [46]. Challenges remain though in identifying the optimal source of antigen for cancer vaccines. Differentiation antigens such as cancer testis antigens have been employed as attractive immunotherapeutic targets based on strong immunogenicity with scant expression on normal tissues [47–49]. Exclusively tumor-specific mutated antigens, while more attractive, are few in number and may not be uniformly expressed across tumors or within an individual tumor [7, 50, 51]. After identification of the target antigen, cancer vaccines must deliver tumor antigens and have been employed to do so in the form of whole tumor lysate, peptides, or nucleic acids [17]. Whole tumor lysate is limited from difficult to access tumors, may include self-antigens, and have been shown to result in poor APC uptake, inadequate antigen cross-presentation, and tepid CD8+ T cell responses [52, 53]. Alternatively, peptide vaccines can be constructed from the most immunogenic or cancer specific epitopes, but since peptides are not sufficiently antigenic, they are typically accompanied by an adjuvant (i.e., GM-CSF, KLH) [54]. Examples of cancer peptide vaccines that have been studied in phase III trials include the MAGE-A3 cancer testis antigen for non-small cell squamous lung cancer/melanoma, rindopepimut for glioblastoma (GBM), and sipuleucel-T for prostate cancer [5, 7, 48, 51]. While cancer testis antigens are promising therapeutic targets, in two phase III trials for non-small cell lung cancer (NSCLC) and melanoma, MAGE-A3 (fusion protein administered with immunostimulant AS15) failed to extend disease-free survival [48]. Similarly, rindopepimut (peptide spanning junction of the tumor-specific EGFRVIII mutation found in 30–40% of GBM patients) failed to meet its prespecified end-point in a randomized phase III study [7, 54]. Sipuleucel-T (a cell based peptide vaccine) activates immature DCs ex vivo using a fusion protein containing prostatic acid phosphatase coupled to GM-CSF and has shown efficacy by improving overall survival in metastatic castration-resistant prostate cancer but remains encumbered by significant cost/complexity, which has prevented its widespread adoption [5, 55]. Meanwhile, the immune correlates of successful vaccination have yet to be fully unraveled [55]. In the case of sipuleucel-T, T cell responses were elevated in the presence of antigen conjugated to GM-CSF and were not as robust using unconjugated antigens highlighting the need for further investigation [55].

Despite limited progress in the setting of therapeutic vaccines, preventative vaccines such as the prophylactic HPV vaccine (Human Papillomavirus 9-valent vaccine, recombinant) have been utilized to avert cervical cancer; however, these vaccines do not induce strong therapeutic responses against established HPV lesions and active infections [56]. While cancers that are virally propagated (i.e., EBV+ Burkitt's disease, hepatocellular carcinoma) may benefit from prophylactic vaccines, new paradigms for therapeutic vaccines need to be established.

Peptide vaccines continue to be mired by local administration of poorly immunogenic antigens [11]. Moreover, peptide vaccines remain limited by MHC class restriction often constraining trials to HLA A2 selected patients (limiting accrual for trials with limited patient numbers (i.e., pediatric malignancies)) [57, 58]. Alternatively, nucleic acids allow for a patient's intracellular machinery to translate and process tumor encoding transcripts based on an individual specific HLA haplotype and can thus be leveraged for the population at large [58–60]. Nucleic acids encoding for tumor antigens can stimulate innate immunity by sensitizing toll-like receptors and intracellular sensors [25]. DNA vaccines are encumbered by having to cross both cell and nuclear membranes with theoretical concerns for genomic integration and oncologic transformation which have paved the way for RNA vaccines [61]. RNA vaccines can activate the innate immune system by acting as TLR agonists for TLR7 and TLR8 [62–64]. As TLRs are activated, innate immunity is initiated through downstream signaling of NF-Kb and production of type I interferons (IFNs) [65]. DAMPs such as heat shock proteins may be incorporated into vaccines to further enhance recruitment of innate immunity while shuttling antigens through the MHC class I pathway to heighten adaptive responses [66, 67]. Our group has shown that preconditioning with tetanus toxoid prior to immunotherapy enhances DC migration to local draining lymph nodes via a CD4+ T cell memory recall response [16, 68–70]. Tetanus boosters mediate recruitment of DCs to draining lymph nodes through a coordinated axis of chemokines including CCL3 and CCL21 mediated in part by these memory T cells [16, 68–70]. These data substantiate the interplay between innate immune activation (via tetanus), migration of DCs, and memory T cells which culminates in enhanced antitumor immunity [16, 68–70]. Recently, systemic RNA delivery encoding for cancer antigens was shown to harness the antiviral defense mechanism [71, 72]. This was shown through systemic IFN alpha dependent activation of APCs and effector cells [71, 72]. The authors propose a mechanism whereby early IFN alpha release from plasmacytoid DCs contributes to migration and maturation of immature DCs while delayed release of IFN alpha from macrophages assists in licensing activated T cells into fully primed effector cells [71, 72]. This cross talk between innate and adaptive immunity is requisite for synergy and was corroborated in a phase I dose escalation trial [71, 72]. In this phase I trial, RNA vaccines encoding for four tumor antigens (NY-ESO-1, MAGE-A3, tyrosinase, and TPTE) were well tolerated and elicited dose-dependent release of early IFN*α* and CXCL10 and de novo T cell immunity against vaccine antigens [71, 72]. In summary, while peptide vaccines have shown tepid responses in a therapeutic setting with poor correlates for immune activity, RNA vaccines activate innate immune responses and have shown promising effects in preclinical and clinical studies based on enhanced DC migration and maturation.

## 4. Immune Checkpoints and Cancer Vaccines

Checkpoint inhibitors are some of the most promising agents that can be exploited to harness the adaptive immune response against cancer antigens [11, 73, 74]. Immune checkpoints can be targeted with monoclonal antibodies (mAbs) targeting cytotoxic T lymphocyte associated antigen-4 (CTLA-4) or programmed death-1 (PD-1) which elicit activation of endogenous T cells against immunogenic epitopes [75]. Ipilimumab, an antagonist of CTLA-4, elicited antitumor T cell responses in a phase III study for patients with metastatic melanoma improving median overall survival by 3.6 months compared with controls [76]. Ipilimumab enhanced immunity against NY-ESO-1, a cancer/testis antigen expressed in some melanoma patients; NY-ESO-1–seropositive patients with CD8+ T cell responses, as compared to patients with undetectable CD8+ T cell responses, experienced greater clinical benefit [3]. Subsequent studies have shown that responsiveness to CTLA-4 is increased in patients with higher burdens of nonsynonymous changes, which may result from endogenous T cell activation against neoantigen epitopes that arise in malignancies with high mutational burdens [77]. Similarly, checkpoint inhibitors antagonizing the PD-1 receptor (nivolumab or pembrolizumab) on T cells have shown remarkable clinical responses in patients with melanoma and lung cancer [78–81]. In patients with advanced nonsquamous non-small cell lung cancer (despite platinum based chemotherapy), the median overall survival was 12.2 months with nivolumab compared to 9.4 months with docetaxel treatment [79]. Like CTLA-4, PD-1 mAbs appear to activate the endogenous T cell response against neoantigens, which may be prospectively identified in the peripheral blood by expression of CD8+PD-1+ lymphocytes [12, 77, 82, 83]. Since anti-PD-1 and CTLA-4 therapies are most effective in patients with evidence for preexisting antitumor immunity, it is likely that these drugs promote established immunity as opposed to inducing de novo responses [84–86]. In a recent study of 46 patients with metastatic melanoma, tumor regression after PD-1 blockade required preexisting CD8+ T cells located at the invasive tumor margin that were negatively regulated by the PD-1 immune inhibitory axis [84]. Since preexisting immunity appears vital for the utility of immune checkpoints, they may have decreased efficacy in cancers with low mutational burdens unless combined with cancer vaccines [87]. Cancer vaccines can be used to generate endogenous immunity against tumor antigens, which can be synergistic with checkpoint inhibitors [88]. Immune checkpoint inhibitors can act upon a nascent immunotherapeutic response initiated by cancer vaccines to sustain their proliferation and viability. In preclinical studies, maximal antitumor efficacy hinged on passive and active vaccination in conjunction with anti-PD-1 blockade [88]. This combination immunotherapy required a T cell vaccine, tumor-targeting-antibody, recombinant IL-2, and anti-PD-1 blockade, which induced recruitment of tumor infiltrating immune cells and production of intratumoral proinflammatory cytokines in a genetically engineered murine melanoma [88].

Interestingly, the antitumor effects of checkpoint blockade seem to be dependent on distinct species of* Bacteroides* [89–93]. In murine models and in patients, T cell responses that were specific against* B. thetaiotaomicron* or* B. fragilis* correlated with the effectiveness from CTLA-4 blockade; however, tumors in antibiotic treated mice did not respond to checkpoint inhibition [92]. In preclinical studies, oral* Bifidobacterium* administration controlled melanoma growth in mice comparably to checkpoint blockade with antiprogrammed death ligand-1 (PD-L1) mAbs [94]. Moreover, the combination of checkpoint blockade and* Bifidobacterium* nearly eliminated outgrowth of tumor [94]. While these effects are peculiar, the augmented effect of PD-L1 mAb is attributed to enhanced dendritic cell (DC) function by intestinal microbes, enabling heightened CD8+ T cell priming and responsiveness to checkpoint blocking strategies [91, 94]. Host-innate immunity in the gut is influenced by paneth cells and intestinal DCs which may be responsible for enhancing T cell immunity induced by checkpoint blockade [95, 96]. Paneth cells are essential for maintaining host microbial homeostasis and directly recognize enteric bacteria through TLR activation by MyD88 [95]. Intestinal DCs induce selective amounts of IgA protecting against mucosal perturbation and can retain limited amounts of live commensals for days [96, 97]. Given this unique interaction between innate gut and adaptive immunity, cancer vaccines may be harnessed to directly enhance DC function for synergy with immune checkpoint inhibition [95–97]. Similar to how commensal bacteria enhance DCs for synergy with checkpoint blockade, cancer vaccines can be leveraged to directly sensitize host APCs [95–97]. The synergy between intestinal bacteria and checkpoint blockade sheds new light on gut microorganisms and their ability to shape host-innate and adaptive immunity which teeter between proinflammatory and regulatory immune responses [10]. While the exact mechanism responsible for interplay between gut microbiota and potentiation of checkpoint blockade needs to be further elucidated, these data implicate the delicate balance between innate and adaptive immunity which might be molded by cancer vaccines to potentiate antitumor immunity [85, 93]. In summary, adaptive immunity is maximized when the innate arm is activated in juxtaposition. Attempts to harness both innate and adaptive immunity have demonstrated promising responses both preclinically and clinically. Identifying methods to unlock effective innate immunity while understating its interplay with adaptive immunity promises to enhance both arms of the immune system.

## 5. Conclusion

Cancer vaccines bypass the complexity of cellular therapeutics and activate the innate immune response against cancer antigens, but many of these platforms suffer from inadequate immunogenicity and lack robust antigen specific T cell responses. Cancer vaccines must overcome the challenges in both their ability to induce the appropriate inflammatory milieu peripherally and their ability to redirect the regulatory stroma intratumorally. To reprogram the peripheral immune milieu, new strategies have been employed to enhance APC recruitment, activation, and trafficking. RNA tumor antigens have been employed to enhance APC recruitment/activation while memory recall responses (using tetanus toxoid) have been shown to enhance APC trafficking. While these advancements appear to directly translate toward improved immunogenicity, potentiating and sustaining these responses remain a significant challenge. To sustain T cell immunity generated by cancer vaccines, there appears to be a synergistic role for cancer vaccines with immune checkpoint blockade, which works through a delicate balance between the innate and adaptive arms of the immune system. Understanding and manipulating the cross talk between both innate and adaptive arms are likely requisite to engender indelible self-sustaining antitumor immunity. As more effective immunotherapeutics make their way from the bench to the bedside, bridging the gap between the innate and adaptive immunity via novel cancer vaccination platforms is vital to prevent competition and allow for maintenance of a sustained response against cancer antigens.
